# Value of ^18^F-FDG PET/CT for predicting axillary pathologic complete response following neoadjuvant systemic therapy in breast cancer patients: emphasis on breast cancer subtype

**DOI:** 10.1186/s13550-021-00861-z

**Published:** 2021-11-22

**Authors:** Cornelis M. de Mooij, Cristina Mitea, Felix M. Mottaghy, Marjolein L. Smidt, Thiemo J. A. van Nijnatten

**Affiliations:** 1 Department of Radiology and Nuclear Medicine, Maastricht University Medical Center+, Maastricht, The Netherlands; 2 Department of Surgery, Maastricht University Medical Center+, P.O. Box 5800, 6202 AZ Maastricht, The Netherlands; 3 GROW – School for Oncology and Developmental Biology, Maastricht University Medical Center+, Maastricht, The Netherlands; 4 Department of Nuclear Medicine, University Hospital RWTH Aachen University, Aachen, Germany

**Keywords:** Positron emission tomography/computed tomography, Neoadjuvant systemic therapy, Breast cancer, Axillary lymph node metastases, Response prediction

## Abstract

**Background:**

Neoadjuvant systemic therapy (NST) is a widely accepted initial treatment modality that can lead to pathologic downstaging of the axillary disease burden in breast cancer patients. Axillary response as well as baseline ^18^F-fluorodeoxyglucose (^18^F-FDG) uptake on positron emission tomography with computed tomography (PET/CT) differ between breast cancer subtypes. The value of baseline ^18^F-FDG PET/CT in predicting axillary response to NST is not yet established, possibly since breast cancer subtype was not taken into account. The purpose of this study was to investigate the value of baseline ^18^F-FDG PET/CT in predicting axillary response to NST with a specific emphasis on subtype.

**Methods:**

PET-parameters derived from the primary tumor as well as the most FDG-avid axillary lymph node were measured on baseline ^18^F-FDG PET/CT. Overall imaging findings were compared with the gold standard of histopathology of the axillary surgery specimen. Analyses for ER-positive/HER2-negative were performed separately from HER2-positive and TN patients. In addition, separate analyses for clinically node-positive patients were performed.

**Results:**

Sixty-six patients with 69 primary tumors were included in this study. Thirty-three axillae contained ER-positive/HER2-negative, 16 HER2-positive, and 20 TN breast cancer. No significant difference in PET-parameters between patients with axillary residual disease and axillary pathologic complete response were found for ER-positive/HER2-negative breast cancer. In the combined HER2-positive/TN subgroup, the SUV_max_ was significantly lower in patients without residual axillary disease in both the entire cohort and in patients with clinically node-positive disease. In this combined subgroup, a cut-off of 4.89 SUV_max_ measured on the most FDG-avid axillary lymph node could predict residual axillary disease with a sensitivity, specificity, PPV, and NPV of 90%, 69%, 53%, and 95%, respectively.

**Conclusions:**

Predicting axillary response following NST with baseline ^18^F-FDG PET/CT can be performed when focusing on breast cancer subtypes. The easily computed PET-parameter SUV_max_ can predict axillary response in HER2-positive and TN breast cancer. This study adds to the accumulating evidence that studies investigating the value of ^18^F-FDG PET/CT in breast cancer should always take subtypes into account.

**Supplementary Information:**

The online version contains supplementary material available at 10.1186/s13550-021-00861-z.

## Background

Neoadjuvant systemic therapy (NST) has become a widely accepted initial treatment modality for breast cancer patients with unfavorable tumor characteristics and/or with axillary lymph node metastases [1, 2]. NST can lead to pathologic downstaging of the axillary disease burden allowing less-invasive axillary surgery [3]. The axillary response to NST is subtype-dependent and patients with human epidermal growth factor receptor 2 positive (HER2-positive) and triple negative (TN) breast cancer are more likely to achieve axillary pathologic complete response (axillary pCR) than patients with estrogen receptor (ER)-positive breast cancer [4]. Importantly, patients with axillary pCR have both improved overall (OS; 85% vs 55%) as well as disease-free survival (DFS; 83% vs 58%) compared to patients with residual axillary disease [5].

Positron emission tomography with computed tomography (PET/CT) using ^18^F-fluorodeoxyglucose (^18^F-FDG) is commonly used to stage patients with locally advanced or recurrent breast cancer [6, 7]. It has been hypothesized that axillary lymph node metastases with higher baseline glycolytic activity, and therefore higher ^18^F-FDG uptake reflected by standardized uptake values (SUVs), achieve axillary pCR less frequently [8, 9]. In this regard, baseline ^18^F-FDG PET/CT prior to NST could contain valuable information regarding axillary response which might aid in the clinical decision making regarding NST or primary surgery. Ideally, a cut-off value of an easily computed PET-parameter, such as maximum SUV (SUV_max_), would be clinically helpful to predict which patients are more likely to achieve axillary pCR following NST [10].

In addition, breast cancer subtype seems to affect the relationship between baseline glycolytic activity on ^18^F-FDG PET/CT and axillary response. While the negative correlation of ER expression with ^18^F-FDG uptake is well established by several studies, the relationship between ^18^F-FDG uptake and HER2 status remains a matter of controversy [11, 12]. However, studies do show that ER-positive/HER2-negative patients often have significantly lower ^18^F-FDG uptake compared to TN and HER2-positive patients, while the difference between TN and HER2-positive patients is less clear [13–16]. The apparent contradiction between higher SUV_max_ in subtypes that tend to respond well to NST indicates that axillary response prediction based on ^18^F-FDG uptake should be investigated by taking breast cancer subtypes into account.

Therefore, the aim of the present study was to determine the value of ^18^F-FDG PET/CT prior to the start of NST to predict which breast cancer patients will achieve axillary pCR following NST with a specific emphasis on breast cancer subtype.

## Methods

### Patient selection

We retrospectively evaluated all female breast cancer patients that underwent an ^18^F-FDG PET/CT exam prior to NST at our facility between 2008 and 2018. Exclusion criteria were the absence of axillary surgery following NST, inflammatory breast cancer and incomplete exams. In all patients, the primary tumor was assessed using mammography, ultrasonography (US), and/or magnetic resonance imaging (MRI). Histological core biopsies of the primary tumor were performed to determine tumor characteristics. The axillary lymph nodes were evaluated with axillary US and concurrent tissue sampling in case of suspicious lymph nodes (i.e., diffuse cortical thickening, focal cortical mass and/or thickening and loss of the fatty hilum) [17]. In patients diagnosed with bilateral invasive breast cancer, lymph nodes were assessed in both axillae separately. The local medical ethics committee waived the necessity to acquire informed consent due to the retrospective study design.

### Treatment

Patients with unfavorable tumor characteristics and/or lymph node metastasis were offered NST at our institution. The type of NST regimens were administered according to the prevailing Dutch national breast cancer guidelines (Additional file 1: Table S1) [18]. The sequential NST regimen generally consisted of four cycles of 3-weekly doxorubicin and cyclophosphamide, followed by either four 3-weekly cycles of docetaxel in case of ER-positive and/or HER2-positive subtype, or by 12 weekly cycles of paclitaxel in case of TN subtype. Moreover, carboplatin could be added in case of TN subtype. Alternatively, patients could be offered a concurrent schedule consisting of six 3-weekly cycles of doxorubicin, cyclophosphamide and docetaxel. In case of HER2-positive subtype, HER2-targeted therapy with trastuzumab and/or pertuzumab was administered following four 3-weekly cycles of doxorubicin and cyclophosphamide. Alternatively, HER2-positive patients could be offered a concurrent schedule consisting of six 3-weekly cycles of docetaxel, trastuzumab and pertuzumab.

Patients with clinically node-negative disease prior to NST underwent a sentinel lymph node biopsy (SLNB). Clinically node-positive patients underwent either an axillary lymph node dissection (ALND) or a combination of the procedure marking axillary lymph nodes with radioactive iodine seeds (MARI) and SLNB [19].

### ^18^F-FDG PET/CT imaging

Prior to the start of NST, patients underwent an ^18^F-FDG PET/CT exam (Gemini TF, Philips Healthcare, Best, the Netherlands) with a standard acquisition protocol [20, 21]. Prior to ^18^F-FDG administration patients had to fast for at least 4 h. Afterward blood glucose levels were checked to ensure levels below 10 mmol/l, and subsequently, an intravenous ^18^F-FDG injection of 2 MBq/kg body weight was administered. A standard supine whole-body ^18^F-FDG-PET/CT with elevated arms was acquired after a resting period of 45–60 min. A low-dose CT scan (120 kV, 30 mAs, slice thickness 4 mm) from head to thigh was performed, followed by the PET acquisition (2 min per bed position). CT images were reconstructed using filtered-back projection. PET images were reconstructed using the BLOB-OS-TF time-of-flight algorithm provided by the manufacturer, with a voxel size of 4 × 4 × 4 mm^3^. The ^18^F-FDG PET/CT imaging protocol did not change during the study period.

### Imaging assessment

**Fig. 1:**
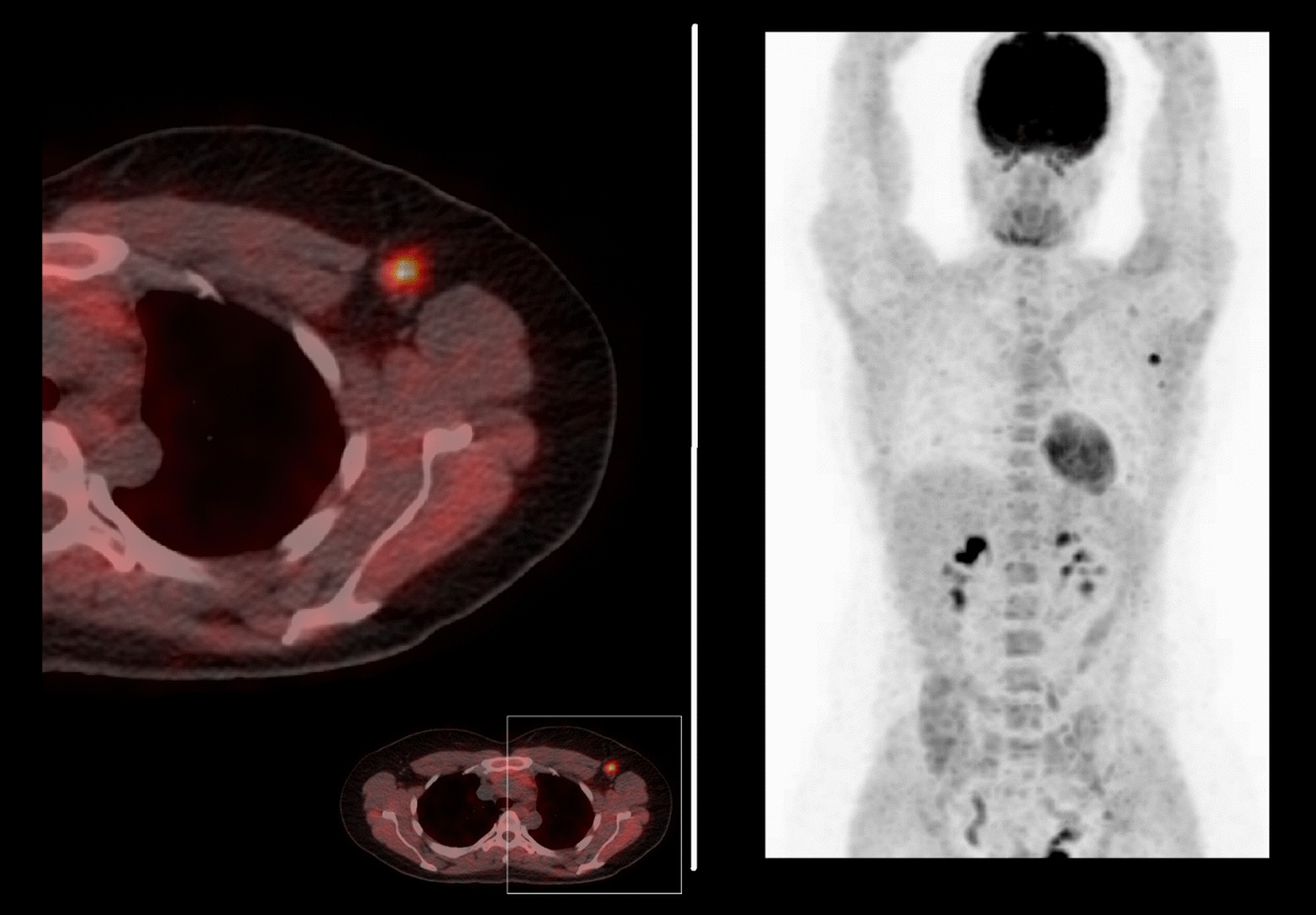
Patient example. Example of a patient with a 22-mm HER2-positive invasive carcinoma of no special type in her left breast. An axial ^18^F-FDG PET/CT exam of the left axilla shows the most FDG-avid axillary lymph node with an SUVmax of 7.37. Following completion NST, this patient had residual axillary disease

A nuclear medicine physician with ten years of experience (C.M.) reviewed all images using simultaneous display of PET, CT, and fused PET/CT images. The reader was blinded for clinicopathologic or follow-up findings other than the presence of breast cancer. All image analyses were performed on a dedicated commercially available workstation (AW-server 3.2, GE Healthcare, Chicago, USA). The ^18^F-FDG uptake in the primary tumor and the most FDG-avid axillary lymph node was semi-quantitatively analyzed using the metabolic PET-parameters maximum, mean and peak SUV (SUV_max_, SUV_mean_, and SUV_peak_) (Fig. 1). SUV-parameters were determined for each region of interest by correcting the measured activity for radioactive decay, total administered activity, and body weight [21]. Moreover, metabolic tumor volume (MTV) was determined by measuring the volume of FDG-avid voxels with an activity equal to or greater than 42% of the SUV_max_ in that specific region of interest and total lesion glycolysis (TLG) was computed by multiplying the MTV with the SUV_mean_. Lastly, the nodal/tumor ratio (NT-ratio) was computed by dividing the SUV_max_ of the most FDG-avid axillary lymph node by the SUV_max_ of the primary tumor [22].

### Response assessment of axillary nodes

Of all axillary surgery specimens, the total number of evaluated lymph nodes and the number of lymph nodes with isolated tumor cells (≤ 0.2 mm or less than 200 cells), micrometastases (> 0.2 and ≤ 2.0 mm), and macrometastases (> 2.0 mm) was reported. Histopathologic response of the axillary lymph nodes and the primary tumor was evaluated according to EUSOMA guidelines and based on reduction of tumor cellularity, using the classification suggested by Pinder et al. [23]. Histopathologic response to NST was based on the axillary lymph node with the most unfavorable category. Axillary pCR was categorized as lymph nodes without metastatic disease and with or without evidence of response/downstaging such as fibrosis, residual axillary disease was defined as lymph nodes with metastatic disease and with or without evidence of response/downstaging such as fibrosis [23].

### Statistical analysis

Statistical analyses were performed using SPSS software (version 25.0, IBM Corporation, Armonk, New York, USA). The difference in response to NST of the axillary region between breast cancer subtypes was compared by use of a Pearsons's chi-squared test. Differences in PET-parameters between patients with and without axillary pCR were examined for statistical significance by the Mann–Whitney U test. Receiver-operating characteristics (ROC) analyses were performed to determine cut-off values of PET-parameters for the prediction of axillary response to NST for PET-parameters that differed significantly between response groups at baseline. Residual axillary lymph node disease was considered positive, and axillary pCR was considered negative. Sensitivity was defined as the proportion of patients with residual axillary disease that were correctly predicted. Specificity was defined as the proportion of patients with axillary pCR that were correctly predicted. Positive predictive value (PPV) was defined as the proportion of patients predicted to have residual axillary disease who had residual axillary disease following NST. Negative predictive value was defined as the proportion of patients predicted to achieve axillary pCR who had axillary pCR following NST. Due to the small sample size in combination with the low incidence of axillary pCR and low ^18^F-FDG uptake in ER-positive/HER2-negative patients, analyses for ER-positive/HER2-negative were performed separately from HER2-positive/TN breast cancer. Additionally, subgroup analysis of clinically node-positive breast cancer patients was performed. All statistical tests were two-sided, with the level of significance established at *P* < 0.05.

## Results

### Patient characteristics

Eighty-one consecutive patients with 87 primary tumors underwent ^18^F-FDG PET/CT prior to NST at the Maastricht University Medical Center between 2008 and 2018. After exclusion of eighteen cases for various reasons [inflammatory breast cancer (*n* = 8), incomplete exams (*n* = 4), no axillary surgery following NST (*n* = 6)], a remaining 66 patients with 69 primary tumors were included in this study. Clinicopathologic and operative characteristics of included patients are listed in Table 1. Of all included patients, 33 had ER-positive/HER2-negative, 16 HER2-positive, and 20 triple-negative (TN) breast cancer. Seventeen axillae were considered clinically node-negative and the remaining 52 axillae were clinically node-positive, based on axillary US findings.

**Table 1:** Clinicopathologic and operative characteristics of all breast cancer patients and subdivided by breast cancer subtype

	ER+/HER2- (*n* = 33)	HER2+ (*n* = 16)	TN (*n* = 20)	Total (*n* = 69)
Age (years, range)				
Median	55 (38–80)	50 (36–68)	52 (37–73)	52 (36–73)
Tumor size (mm, range)				
Median	33 (13–90)	39 (8–87	45 (11–77)	41 (8–90)
cT status (*n*, %)				
cT1	2 (6.3%)	1 (6.3%)	1 (5.0%)	4 (5.9%)
cT2	20 (62.5%)	10 (62.5%)	10 (50.0%)	40 (58.8%)
cT3	8 (25.0%)	5 (31.3%)	9 (45.0%)	22 (32.4%)
cT4	2 (6.3%)	0 (0.0%)	0 (0.0%)	2 (2.9%)
cN status (*n*, %)				
cN0	7 (21.2%)	4 (25.0%)	4 (20.0%)	15 (21.7%)
cN1	22 (66.7%)	6 (37.5%)	11 (55.0%)	39 (56.5%)
cN2-3	4 (12.1%)	6 (37.5%)	5 (25.0%)	15 (21.7%)
PET-positive nodes (*n*, %)				
0	10 (30.3%)	5 (31.3%)	4 (20.0%)	19 (27.5%)
1–3	15 (45.5%)	9 (56.3%)	11 (55.0%)	35 (50.7%)
≥ 4	8 (24.2%)	2 (12.5%)	5 (25.0%)	15 (21.7%)
Histology (*n*, %)				
Invasive NST	31 (93.9%)	14 (87.5%)	18 (90.0%)	63 (91.3%)
ILC	2 (6.1%)	1 (6.3%)	0 (0.0%)	3 (4.3%)
Other	0 (0.0%)	1 (6.3%)	2 (10.0%)	3 (4.3%)
ER (*n*, %)				
Negative	0 (0.0%)	5 (31.3%)	20 (100.0%)	25 (36.2%)
Positive	33 (100.0%)	11 (68.8%)	0 (0.0%)	44 (63.8%)
PR (*n*, %)				
Negative	12 (36.4%)	10 (62.5%)	20 (100.0%)	42 (60.9%)
Positive	21 (63.6%)	6 (37.5%)	0 (0.0%)	27 (39.1%)
Tumor grade (*n*, %)				
Grade 1	4 (12.1%)	2 (12.5%)	0 (0.0%)	6 (8.7%)
Grade 2	18 (54.5%)	5 (31.3%)	4 (20.0%)	27 (39.1%)
Grade 3	11 (33.3%)	9 (56.3%)	16 (80.0%)	36 (52.2%)
Focality (*n*, %)				
Unifocal	22 (66.7%)	11 (68.8%)	11 (55.0%)	44 (63.8%)
Multifocal	11 (33.3%)	5 (31.3%)	9 (45.0%)	25 (36.2%)
Breast surgery (*n*, %)				
Lumpectomy	10 (31.3%)	5 (31.3%)	6 (30.0%)	21 (30.9%)
Mastectomy	22 (68.8%)	11 (68.8%)	14 (70.0%)	47 (69.1%)
Axillary surgery (*n*, %)				
SLNB	8 (24.2%)	4 (24.2%)	5 (25.0%)	17 (24.6%)
ALND	24 (72.7%)	11 (68.8%)	12 (60.0%)	47 (68.1%)
SLNB/MARI	1 (3.0%)	1 (6.3%)	3 (15.0%)	5 (7.2%)

ALND, axillary lymph node dissection; BR, Bloom-Richardson; cN, clinical nodal; cT, clinical tumor; ER, estrogen receptor; HER2, human epidermal growth factor receptor 2; ILC, invasive lobular carcinoma; MARI, marking the axilla with radioactive iodine seeds; NST, no specific type; PET, positron emission tomography; PR, progesterone receptor; SLNB, sentinel lymph node biopsy; TN, triple negative

### Axillary response to NST

Overview of the pathologic response of the axillary lymph nodes to NST is displayed in Table 2. When considering all patients there was no evidence of axillary residual disease following NST in 37 axillae (53.6%). In the subgroup of clinically node-positive breast cancer patients axillary pCR was achieved in a total of 25 axillae (48.1%).

**Table 2:** Axillary response following NST

Characteristic	All patients (*n* = 69)	ER+/HER2- (*n* = 33)	HER2+ (*n* = 16)	TN (*n* = 20)	*p*-value
*All patients*					
Axillary response					0.001^a^
Axillary residual	32 (46.4%)	22 (66.7%)	2 (12.5%)	8 (40.0%)	
No axillary residual	37 (53.6%)	11 (33.3%)	14 (87.5%)	12 (60.0%)	
*Clinically node-positive patients*					
Axillary response					0.014^a^
Axillary residual	27 (51.9%)	17 (68.0%)	2 (16.7%)	8 (53.3%)	
Axillary pCR	25 (48.1%)	8 (32.0%)	10 (83.3%)	7 (46.7%)	

ER, estrogen receptor; HER2, human epidermal growth factor 2 receptor; pCR, pathologic complete response

^a^Pearsons’ chi-square test; all variables are displayed as number and percentage

When considering all patients, there is a significant difference (*p* < 0.01) in axillary response after NST between subtypes with the highest percentage of patients without axillary residual disease in HER2-positive breast cancer (87.5%), followed by the TN (60.0%) and ER-positive/HER2-negative subtypes (33.3%). In the subgroup of clinically node-positive breast cancer patients there is a consistent significant difference (*p* = 0.01) in axillary pCR between subtypes with the highest percentage in HER2-positive breast cancer (83.3%), followed by the TN (46.7%) and ER-positive/HER2-negative subtypes (32.0%).

### PET-parameters associated with axillary response to NST

The NT-ratio (0.75 vs 0.39, *p* = 0.025) is the only PET-parameter for which a significant difference is reported between response groups regarding the whole cohort (Table 3). Similar analyses were performed for a combined cohort of HER2-positive/TN breast cancer. In this subgroup, significant differences between presence and absence of axillary residual disease following NST were reported for the PET-parameters SUV_max_ (7.50 vs 3.15, *p* = 0.002), SUV_mean_ (4.69 vs 2.07, *p* = 0.002), SUV_peak_ (5.69 vs 2.66, *p* = 0.007), TLG (7.77 vs 3.46, *p* = 0.028) and NT ratio (1.18 vs 0.39, *p* = 0.008), with higher values found for patients with residual axillary disease. The difference is consistently significant in the subgroup of clinically node-positive HER2-positive/TN patients for SUV_max_ (7.50 vs 4.53, *p* = 0.040) and SUV_mean_ (4.69 vs 3.01, *p* = 0.031). For the subgroup of patients with ER-positive/HER2-negative breast cancer, none of the measured PET-parameters differed between axillary response groups.

**Table 3:** Differences in PET-parameters determined on the most FDG-avid axillary lymph node between axillary response groups

	All patients			All cN+ patients		
	Axillary residual (*n* = 32)	No axillary residual (*n* = 37)	*p*-value	Axillary residual (*n* = 27)	Axillary pCR (*n* = 25)	*p*-value
*All patients*						
SUV_max_	5.09 0.56–17.63	3.43 0.64–18.67	0.126^a^	5.86 1.17–17.63	5.28 1.51–18.67	0.301
SUV_mean_	3.33 0.56–11.01	2.00 0.58–12.20	0.134^a^	3.84 0.75–11.01	3.29 1.11–12.20	0.276
SUV_peak_	4.10 0.78–12.57	2.75 0.60–16.17	0.121^a^	4.42 0.78–12.57	3.73 1.44–16.17	0.451
MTV	1.70 0.51–15.74	1.73 0.19–9.47	0.947^a^	1.98 0.51–15.74	2.30 0.45–9.47	0.755
TLG	6.41 0.36–120.88	3.74 0.12–115.53	0.336^a^	7.26 1.16–120.88	8.92 0.64–115.53	0.791
NT-ratio	0.75 0.18–2.75	0.39 0.07–5.05	**0.025**^**a**^	0.88 0.18–2.75	0.83 0.27–5.05	0.147
*HER2-positive or TN patients*						
SUV_max_	7.50 1.66–17.63	3.15 0.64–18.67	**0.002**^**a**^	7.50 1.66–17.63	4.53 2.35–18.67	**0.040**^**a**^
SUV_mean_	4.69 1.23–11.01	2.07 0.58–12.20	**0.002**^**a**^	4.69 1.23–11.01	3.01 1.46–12.20	**0.031**^**a**^
SUV_peak_	5.69 1.17–12.57	2.66 0.60–16.17	**0.007**^**a**^	5.69 1.17–12.57	3.42 1.52–16.17	0.053^a^
MTV	1.70 1.09–15.74	1.89 0.19–9.47	0.821^a^	1.70 1.09–15.74	2.18 0.45–9.47	0.639^a^
TLG	7.77 1.57–120.88	3.46 0.12–115.53	**0.028**^**a**^	7.77 1.57–120.88	5.21 0.89–115.53	0.309^a^
NT-ratio	1.18 0.18–2.47	0.39 0.11–2.74	**0.008**^**a**^	1.18 0.18–2.47	0.56 0.27–2.74	0.083^a^
*ER*+*/HER2- patients*						
SUV_max_	3.90 0.56–10.23	3.52 1.27–11.34	0.807^a^	4.70 1.17–10.23	5.38 1.51–11.34	0.887^a^
SUV_mean_	2.50 0.56–6.71	2.00 1.00–7.21	0.807^a^	3.20 0.75–6.71	3.41 1.11–7.21	0.887^a^
SUV_peak_	2.97 0.78–7.88	3.15 1.01–8.36	0.792^a^	3.18 0.78–7.88	4.21 1.44–8.36	0.535^a^
MTV	1.79 0.51–6.98	1.73 0.58–8.90	0.611^a^	2.24 0.51–6.98	2.69 0.58–8.90	0.711^a^
TLG	4.72 0.36–39.51	4.05 0.64–37.11	0.693^a^	7.17 1.16–39.51	10.36 0.64–37.11	0.588^a^
NT-ratio	0.64 0.18–2.75	0.46 0.07–5.05	0.848^a^	0.85 0.24–2.75	1.04 0.39–5.05	0.759^a^

Bold* p*-value indicates significance

FDG, ^18^F-fluorodeoxyglycose; HER2, human epidermal growth factor 2 receptor; TN, triple negative MTV, metabolic tumor volume; NT-ratio, nodal tumor ratio; pCR, pathologic complete response; SUV, standardized uptake values; TLG, total lesion glycolysis

^a^Mann-Whitney U test; all variables are displayed as median and range

For the whole cohort as well for the subtypes separately, none of the PET-parameters determined on the primary tumor prior to NST was significantly associated with axillary response following NST (Additional file 1: Table S2).

### Overall predictive value of PET-parameters for residual axillary disease

Regarding the entire cohort of HER2-positive/TN breast cancer patients, the ROC curve for baseline SUV_max_ and baseline SUV_mean_ showed an AUC of 0.82 and 0.83, respectively (Additional file 1: Table S3). In the subgroup of clinically node-positive HER2-positive/TN breast cancer patients, the AUCs were 0.74 and 0.75, respectively.

In the entire cohort of HER2-positive/TN breast cancer, the highest diagnostic accuracy to predict axillary pCR based on SUV_max_ was achieved by using a cut-off of 4.89 on the most FDG-avid axillary lymph node, yielding a sensitivity, specificity, PPV, and NPV of 90%, 69%, 53%, and 95%, respectively (Table 4).

**Table 4:** ROC analyses of PET-parameters in predicting axillary response following NST in HER2-positive/TN breast cancer patients

	AUC	Cut-off	Sensitivity (%)	Specificity (%)	PPV (%)	NPV (%)
*All HER2-positive/TN breast cancer patients*						
LN–SUV_max_	0.82 (0.67–0.98)	4.89	90 (54–99)	69 (48–85)	53 (29–76)	95 (72–100)
LN–SUV_mean_	0.83 (0.67–99)	3.77	90 (54–99)	81 (60–93)	64 (36–86)	96 (75–100)
*Clinically node-positive HER2-positive/TN breast cancer patients*						
LN–SUV_max_	0.74 (0.53–0.95)	7.07	70 (35–92)	82 (56–95)	70 (35–92)	82 (56–95)
LN–SUV_mean_	0.75 (0.54–0.96)	3.77	90 (54–99)	71 (44–89)	64 (36–71)	92 (62–100)

AUC, area under the curve; HER2, human epidermal growth factor 2 receptor; LN, lymph node; NPV, negative predictive value; PPV, positive predictive value; SUV, standardized uptake value; TN, triple negative

## Discussion

This study demonstrates that focusing on breast cancer subtype allows for the prediction of axillary response after completion NST using easily computed PET-parameters. A cut-off of 4.89 SUV_max_ measured on the most FDG-avid axillary lymph node in breast cancer patients with the HER2-positive or TN subtype achieved fair diagnostic accuracy with an AUC of 0.82 in predicting axillary response following NST. Specifically, a SUV_max_ lower than 4.89 measured on the most FDG-avid axillary lymph node in breast cancer patients with the HER2-positive or TN subtype is predictive of having no residual axillary disease following NST with an NPV of 95%.

Molecular subtypes based on expression of receptors strongly influence prognosis and therapeutic approach [24, 25]. Similar to literature, axillary pCR occurred more frequently in HER2-positive and TN than in ER-positive/HER2-negative patients [26]. The rates of axillary pCR found in this study are in line with previously reported rates of axillary pCR in the HER2-positive and TN breast cancer subtypes [27–31]. The rate of axillary pCR in the ER-positive/HER2-negative subtype in our study is strikingly high when compared to previous studies which can possibly be explained by the low sample size of this study [10]. Since the HER2-positive and TN subtypes are more likely to achieve axillary pCR, prediction of axillary pCR seems clinically more relevant in these subtypes [4].

The association between breast cancer subtype and ^18^F-FDG uptake has been extensively investigated. Similar to many previous studies, we report a clear trend with the highest ^18^F-FDG uptake in the TN subtype, followed by HER2-positive and ultimately ER-positive/HER2-negative [13, 14, 16, 32–34]. Not all PET-parameters differed significantly between subtypes, possibly owing to the small sample size of this study. Another explanation can be that the SUV_mean_ provides a better representation of the heterogeneity in the primary tumor compared to SUV_max_ or SUV_peak_, which is clearly shown by the smaller ranges reported for SUV_mean_. The wide and mostly overlapping ranges between breast cancer subtypes can be explained by the fact that the molecular subtypes do not perfectly represent the true diversity and metabolic heterogeneity of breast cancer [35, 36]. However, especially studies with larger sample sizes do show a clear effect of the expression of ER and HER2 on ^18^F-FDG uptake [16, 33, 34, 37–39]. This study adds to the accumulating evidence regarding the differences in ^18^F-FDG uptake between subtypes indicating that future research in this field should always take breast cancer subtype into account.

Baseline differences in PET-parameters between response groups measured on axillary lymph node metastases have been investigated before. Keam et al. did not find a difference in baseline SUV_max_ between clinically node-positive patients with axillary pCR and residual axillary disease [40]. Similar to the results reported in this paper, Rousseau et al. did find that the SUV_max_ was lower in patients that developed axillary pCR following NST [9]. Akdeniz et al. investigated baseline differences in baseline SUV_max_ on axillary lymph node metastases in breast cancer subtypes, but did not report any significant differences [41]. With regard to HER2-positive and TN breast cancer, we do report statistically significant differences in various PET-parameters between patients with axillary pCR and residual axillary disease. The parameters SUV_max_ and SUV_mean_ measured on the most FDG-avid axillary lymph node are persistently lower in HER2-positive and TN patients that develop axillary pCR following NST. Accordingly, these parameters can be investigated for their added value to predict axillary response following NST in clinical practice.

Despite studies reporting on baseline differences in SUV_max_ between axillary pCR and residual axillary disease, its value in predicting axillary response to NST could not yet be established previously [8, 41, 42]. A possible explanation for this low diagnostic accuracy is that previous studies did not focus on subtypes when predicting axillary response with baseline ^18^F-FDG PET/CT. We found that focusing on the HER2-positive and TN subtype could increase the AUC to 0.82 at a cut-off of 4.89 SUV_max_ [8, 42]. Further focusing on the subgroup of clinically node-positive HER2-positive and TN breast cancer patients, the AUC decreased slightly to 0.74 at a cut-off of 3.77 SUV_max_ measured on the most-FDG avid axillary lymph node. An SUV_max_ lower than 3.77 was able to reliably exclude axillary residual disease with an NPV of 92.3%. While the AUCs for SUV_mean_ were consistently higher than those found for SUV_max_, computing the SUV_mean_ is prone to more inter- and intraobserver variability and therefore not applicable in daily clinical practice [43].

The value of sequential ^18^F-FDG PET/CT for the early prediction of axillary response to NST has also been previously explored. Three studies reported a significant increase in diagnostic performance when percentage decrease after the first cycle of NST was used to distinguish between axillary response groups [8, 9, 42]. Interestingly, two studies reported an increase in performance when focusing on specific breast cancer subtypes [8, 42]. Contrarily to our results, Wu et al. reported improved predictive value when excluding ER-negative/HER2-positive patients. A possible explanation could be that ER-positive/HER2-negative patients were underrepresented in the final analysis since patients with a baseline SUV_max_ of the most FDG-avid axillary lymph node < 2.5 were excluded from further analysis [42]. Nevertheless, these previous results as well as the findings of our research indicate that breast cancer subtype should be taken into account when using ^18^F-FDG uptake for axillary response prediction.

Large early trials comparing NST with adjuvant systemic therapy (AST) in breast cancer found no significant difference in DFS or OS, permitting the use of NST for its advantages in allowing less invasive surgery [44–47]. However, these early trials were not specifically aimed at molecular subtypes. A recent systematic review of 9 studies including 36,480 TN breast cancer patients showed that developing a pCR provides a significant advantage in OS and DFS, with hazard ratios of 0.53 (0.29–0.98) and 0.52 (0.29–0.94), respectively, compared to AST in this subtype [48]. Contrarily, having residual disease in the TN subtype deteriorates OS and DFS, with hazard ratios of 1.19 (1.09–1.28) and 2.36 (1.42–3.89), respectively, suggesting that these patients would have benefited from primary surgery followed by AST [48]. Accordingly, TN breast cancer patients more likely not to respond to NST could benefit from earlier tumor debulking with a decreased opportunity for systemic tumor seeding and micrometastases [49]. These data suggest that in the TN subtype predicting response to NST could provide valuable information for selecting patients more suited for primary surgery followed by AST, potentially based on ^18^F-FDG PET/CT findings.

Besides identification of residual axillary disease following NST, prediction of axillary pCR is equally clinically relevant. To date, current noninvasive imaging techniques remain inaccurate to reliably detect which patients have developed an axillary pCR following NST [50]. Meanwhile, less-invasive axillary surgical staging techniques such as SLNB and MARI, performed separately or combined in the TAD- or RISAS-procedures, are investigated and gaining support to omit further axillary treatment [19, 51, 52]. Accordingly, less-invasive axillary surgery could be harmful in patients with residual axillary disease with higher chances of metastatic dissemination. Therefore, it is paramount to investigate noninvasive imaging modalities that can reliably predict or detect axillary response and thus select patients for less-invasive axillary surgery.

This study has several limitations. First, due to the small sample size per subtype HER2-positive and TN breast cancer patients were analyzed combined. Preferably, analyses are performed in large numbers of patients per subtype. Moreover, we did not perform a logistic regression analysis to determine confounding factors associated with subtype as well as with pathologic response to NST because of the small sample size. Second, the inclusion of patients is over a long period of time during which the neoadjuvant regimens have changed, thus influencing the rate of axillary pCR in especially the HER2-positive subtype. Third, this single-center, single-vendor study might limit the external validity of this research since the use of different PET/CT systems or settings might influence the absolute values of PET-parameters. However, using the NT-ratio could possibly overcome this limitation since it is not dependent of the individual PET-parameters.

A focus of future research could be on identifying breast cancer subgroups in which response prediction can be reliably performed. Additionally, the emerging modality ^18^F-FDG PET/MRI could further improve the diagnostic performance of noninvasive imaging in predicting or detecting axillary response to NST in breast cancer. Recent studies have shown promising results of ^18^F-FDG PET/MRI in breast cancer and have suggested it could potentially function as a one-stop-shop solution for patients in need of locoregional as well as distant staging [53–55]. Combining morphologic MRI parameters with metabolic PET-parameters of sequential ^18^F-FDG PET/MRI has shown promising results in predicting primary tumor response in breast cancer in two previous studies [56, 57]. Lastly, major advances in artificial intelligence could further increase the efficiency and accuracy of the prediction and detection of nodal response with imaging [58].

## Conclusions

To conclude, this study was the first to demonstrate that predicting axillary response to NST with baseline ^18^F-FDG PET/CT can be performed when focusing on breast cancer subtype. The parameters SUV_max_ and SUV_mean_ can predict axillary response in HER2-positive and TN breast cancer patients with fair diagnostic accuracy in the entire cohort as well as in clinically node-positive patients. Baseline ^18^F-FDG PET/CT can be valuable in selecting patients more suited for either primary surgery followed by AST or for NST prior to surgery.

## Supplementary Information

**Additional file 1: Table S1.** Administered NST regimens per breast cancer subtype. **Table S2.** Differences in PET-parameters determined on primary tumor between axillary response groups. **Table S3.** ROC analyses of PET-parameters in predicting axillary response following NST in HER2-positive/TN breast cancer patients.

## Data Availability

Raw data were generated at Maastricht University Medical Center. The authors confirm that the data supporting the findings of this study are available within the article and its supplementary analysis. Data and analyses are available from the corresponding author on reasonable request. The code used for data cleaning and analysis is available from the corresponding author on request.

## Abbreviations

^18^F-FDG: ^18^F-fluorodeoxyglucose
ALND: Axillary lymph node dissection
AST: Adjuvant systemic therapy
AUC: Area under the curve
DFS: Disease-free survival
ER: Estrogen receptor
HER2: Human epidermal growth factor receptor 2
MARI: Marking axillary lymph nodes with radioactive iodine seeds
MRI: Magnetic resonance imaging
MTV: Metabolic tumor volume
NPV: Negative predictive value
NST: Neoadjuvant systemic therapy
NT-ratio: Nodal/tumor ratio
OS: Overall survival
PET/CT: Positron emission tomography/computed tomography
pCR: Pathologic complete response
PPV: Positive predictive value
ROC: Receiver operating characteristic
SLNB: Sentinel lymph node biopsy
SUV: Standardized uptake value
TLG: Total lesion glycolysis
TN: Triple negative
US: Ultrasonography
