# Supplementary material for: Value of 18F-FDG PET/CT for predicting axillary pathologic complete response following neoadjuvant systemic therapy in breast cancer patients: emphasis on breast cancer subtype
Source: EJNMMI Res. 2021 Nov 22;11:116. doi: 10.1186/s13550-021-00861-z (PMC8609064; doi:10.1186/s13550-021-00861-z)
Supplement: Supplementary file 1 — Additional file 1: Table S1. Administered NST regimens per breast cancer subtype. Table S2. Differences in PET-parameters determined on primary tumor between axillary response groups. Table S3. ROC analyses of PET-parameters in predicting axillary response following NST in HER2-positive/TN breast cancer patients. [file 13550_2021_861_MOESM1_ESM.docx]

### Additional Table 1. Administered NST regimens per breast cancer subtype.

| **Subtype** | **Regimen** |
| --- | --- |
| **ER+/HER2-**  (n=33) | ***AC-docetaxel (n=24)*** Four 3-weekly cycles of doxorubicin and cyclophosphamide followed by four 3-weekly cycles of docetaxel.  ***TAC (n=8)***  Six 3-weekly cycles of docetaxel, doxorubicin and cyclophosphamide.  ***Docetaxel and cyclophosphamide (n=1)***  Four 3-weekly cycles of docetaxel and cyclophosphamide. |
| **HER2+**  (n=16) | ***AC-docetaxel and trastuzumab (n=7)***  Four 3-weekly cycles of doxorubicin and cyclophosphamide followed by twelve weekly cycles of docetaxel and trastuzumab.  ***AC-docetaxel and trastuzumab (n=5)***  Four 3-weekly cycles of doxorubicin and cyclophosphamide followed by twelve weekly cycles of docetaxel and trastuzumab.  ***Docetaxel, trastuzumab and pertuzumab (n=3)***  Six 3-weekly cycles of docetaxel, trastuzumab and pertuzumab.  ***Carboplatin, paclitaxel, trastuzumab and pertuzumab (n=1)***  Nine 3-weekly cycles of carboplatin, paclitaxel, trastuzumab and pertuzumab. |
| **TN**  (n=20) | ***AC-paclitaxel (n=15)*** Four 3-weekly cycles of doxorubicin and cyclophosphamide followed by twelve weekly cycles of paclitaxel.  ***TAC (n=4)***  Six 3-weekly cycles of docetaxel, doxorubicin and cyclophosphamide.  ***Carboplatin and paclitaxel-AC (n=1)***  Four 3-weekly cycles of carboplatin and paclitaxel followed by four 2-weekly cycles of doxorubicin and cyclophosphamide. |

### AC, doxorubicin and cyclophosphamide; ER, estrogen receptor; HER2, human epidermal growth factor 2 receptor; TAC, docetaxel, doxorubicin and cyclophosphamide; TN, triple negative.

### Additional Table 2. Differences in PET-parameters determined on primary tumour between axillary response groups.

|  | **All patients** | | | **All cN+ patients** | | |
| --- | --- | --- | --- | --- | --- | --- |
|  | **Axillary residual *(n=32)*** | **No axillary residual *(n=37)*** | **P-value** | **Axillary residual**  **(*n = 27*)** | **Axillary pCR**  **(*n = 25*)** | **P-value** |
| ***All patients*** | | | | | | |
| **SUV_max_** | 4.87 1.86-20.81 | 6.22 1.62-19.95 | 0.440^a^ | 5.16  1.86-20.81 | 6.47  1.62-19.63 | 0.869 |
| **SUV_mean_** | 3.02  1.11-13.80 | 3.73  0.92-13.04 | 0.369^a^ | 3.04  1.25-13.80 | 3.74  0.92-13.04 | 0.748 |
| **SUV_peak_** | 4.01  1.61-17.64 | 4.19  1.23-15.68 | 0.453^a^ | 4.18  1.70-17.64 | 4.21  1.23-15.68 | 0.755 |
| **MTV** | 9.09  0.78-55.49 | 6.21  1.22-45.82 | 0.286^a^ | 9.60  0.78-55.49 | 7.42  1.73-45.82 | 0.946 |
| **TLG** | 23.73  1.39-179.05 | 20.87  2.54-365.33 | 0.744^a^ | 23.92  1.39-179.05 | 26.59  4.83-106.76 | 0.793 |
| ***HER2-positive or TN patients*** | | | | | | |
| **SUV_max_** | 9.19  3.08-19.98 | 6.52  2.83-19.63 | 0.566^a^ | 9.19  3.08-19.98 | 7.29  2.83-19.63 | 0.749^a^ |
| **SUV_mean_** | 6.01  1.78-13.80 | 3.96  1.53-13.04 | 0.543^a^ | 6.01  1.78-13.80 | 4.52  1.69-13.04 | 0.786^a^ |
| **SUV_peak_** | 7.59  2.07-17.64 | 5.78  1.64-15.68 | 0.706^a^ | 7.59  2.07-17.64 | 5.79  2.08-15.68 | 0.863^a^ |
| **MTV** | 9.73  0.78-23.81 | 7.55  1.22-45.82 | 0.741^a^ | 9.73  0.78-23.81 | 7.68  1.73-45.82 | 1.000^a^ |
| **TLG** | 71.01  1.39-179.05 | 25.47  2.54-365.33 | 0.590^a^ | 71.01  1.39-179.05 | 37.30  7.13-106.76 | 0.824^a^ |
| ***ER+/HER2- patients*** | | | | | | |
| **SUV_max_** | 4.57  1.86-20.81 | 5.04  1.62-19.95 | 0.685^a^ | 4.81  1.86-20.81 | 4.84  1.62-6.09 | 0.658^a^ |
| **SUV_mean_** | 2.67  1.11-13.47 | 3.13  0.92-12.57 | 0.623^a^ | 2.93  1.25-13.47 | 2.81  0.92-3.73 | 0.609^a^ |
| **SUV_peak_** | 3.41  1.61-16.58 | 3.86  1.23-14.69 | 0.881^a^ | 3.70  1.70-16.58 | 3.61  1.23-4.18 | 0.562^a^ |
| **MTV** | 8.10  1.22-55.49 | 5.25  2.69-20.80 | 0.236^a^ | 6.59  1.22-55.49 | 5.73  2.69-20.80 | 0.708^a^ |
| **TLG** | 21.63  7.74-161.53 | 18.77  4.83-56.58 | 0.334^a^ | 19.36  7.74-161.53 | 14.94  4.83-56.58 | 0.319^a^ |

*^a^ Mann-Whitney U test; all variables are displayed as median and range; FDG, ^18^F-fluorodeoxyglycose; HER2, human epidermal growth factor 2 receptor; MTV, metabolic tumor volume; NT-ratio, nodal tumor ratio; pCR, pathologic complete response; SUV, standardized uptake values; TLG, total lesion glycolysis; TN, triple negative.*

***Additional Table 3. ROC analyses of PET-parameters in predicting axillary response following NST in HER2-positive/TN breast cancer patients.***

| ***Parameter*** | ***Axillary residual*** | ***Axillary pCR*** | ***AUC*** | ***Sensitivity (%)*** | ***Specificity (%)*** | ***Cut-off*** |
| --- | --- | --- | --- | --- | --- | --- |
| ***All HER2-positive or TN patients*** | | | | | | |
| **SUV_max_** | 10 | 26 | 0.82 (0.67-0.98) | 90 (54-99) | 69 (48-85) | 4.89 |
| **SUV_mean_** | 10 | 26 | 0.83 (0.67-0.99) | 90 (54-99) | 81 (60-93) | 3.77 |
| **SUV_peak_** | 10 | 22 | 0.80 (0.62-0.97) | 80 (44-96) | 77 (56-90) | 4.04 |
| **MTV** | 10 | 26 | 0.53 (0.32-0.73) | 100 (66-100) | 23 (10-44) | 1.06 |
| **TLG** | 10 | 26 | 0.74 (0.56-0.91) | 80 (44-96) | 69 (48-72) | 5.35 |
| ***NT-ratio*** | 10 | 26 | 0.79 (0.60-0.97) | 70 (35-92) | 81 (60-93) | 0.88 |
| ***Clinically node-positive HER2-positive or TN patients*** | | | | | | |
| **SUV_max_** | 10 | 17 | 0.74 (0.53-0.95) | 70 (35-92) | 82 (56-95) | 7.07 |
| **SUV_mean_** | 10 | 17 | 0.75 (0.54-0.96) | 90 (54-99) | 71 (44-89) | 3.77 |
| **SUV_peak_** | 10 | 16 | 0.73 (0.52-0.95) | 80 (44-96) | 69 (41-88) | 4.04 |
| **MTV** | 10 | 17 | 0.44 (0.22-0.67) | 100 (66-100) | 12 (2-38) | 0.99 |
| **TLG** | 10 | 17 | 0.62 (0.40-0.85) | 80 (44-96) | 53 (29-76) | 5.35 |
| ***NT-ratio*** | 10 | 17 | 0.71 (0.48-0.93) | 50 (20-80) | 94 (69-100) | 1.28 |

*AUC, area under the curve; FDG, ^18^F-fluorodeoxyglycose; HER2, human epidermal growth factor 2 receptor; LN, lymph node; NPV, negative predictive value; PPV, positive predictive value; SUV, standardized uptake value; TN, triple negative; YI, Youden’s index.*
